# How end-of-life care was limited during the first 18 months of the COVID-19 pandemic: a longitudinal survey study among healthcare providers (the CO-LIVE study)

**DOI:** 10.1186/s12904-024-01514-3

**Published:** 2024-07-26

**Authors:** Masha S. Zee, H. Roeline Pasman, Erica Witkamp, Anne Goossensen, Ida J. Korfage, Yvonne N. Becqué, Corine Nierop-van Baalen, Agnes van der Heide, Bregje D. Onwuteaka-Philipsen

**Affiliations:** 1 Department of Public and Occupational Health, Expertise Center for Palliative Care, Amsterdam UMC, VU University, Van Der Boechorststraat 7, Amsterdam, 1081 BT the Netherlands; 2 Department of Public Health, Erasmus MC University Medical Center, Rotterdam, The Netherlands; 3 Research Center Innovations in Care, Rotterdam University of Applied Sciences, Rotterdam, The Netherlands; 4 University of Humanistic Studies, Utrecht, The Netherlands

**Keywords:** COVID-19, End of life care, Quality of care, Health care providers

## Abstract

**Background:**

During the COVID-19 pandemic, the way in which end-of-life care was provided, underwent a lot of changes and therefor different domains of end-of-life care were impacted. The aim of this study is to describe whether health care providers considered end-of-life care (in medical, nursing, psychosocial and spiritual care) limited by the pandemic through the first 18 months of the COVID-19 pandemic, and examine associations with COVID-19 related circumstances of care (e.g. visit restrictions) and health care providers’ characteristics.

**Methods:**

A longitudinal survey study among healthcare providers from different healthcare settings who provided end-of-life care during the pandemic’s first 18 months. Data of four time periods were analyzed using descriptive statistics and Generalized Estimating Equation.

**Results:**

Of the respondents (*n* = 302) the majority had a nursing background (71.8%) and most worked in a hospital (30.3%). Especially in the first wave end-of-life care in all aspects was limited according to a substantial part of health care providers (between 29.7 and 57.7%). Psychosocial and spiritual care were more limited than medical and nursing care during all time periods. Care being limited according to health care providers was associated with visit restrictions, shortness of personal protective equipment or restrictions in caring for the deceased and decreased over time.

**Conclusion:**

The COVID-19 pandemic impacted different aspects of end-of-life care throughout the pandemic’s first 18 months. Over the course of the pandemic health care providers seemed to have invented ways to adjust their work in order to minimize the effect of limiting measures. More involvement of health care providers in decision-making may improve the prioritization of measures to deal with crisis situations in care. These reflections highlight priorities during crises and the role healthcare providers could play in maintaining good end-of-life care. This remains relevant in new health crises, where care may differ from what is considered good quality of care.

## Introduction

During the COVID-19 pandemic, the way in which end-of-life care was provided, underwent a lot of changes, for both patients with and without COVID-19. Good end-of-life care consists of care within the physical, psychological, social and spiritual domains and includes the support for the loved ones of the patient [1]. However, care in these domains was often impacted due to the consequences of the pandemic, including uncertainty on how to manage the virus, high work pressure and care restrictions. High work pressure for instance was related to an influx of COVID-19 patients and sickness absence of healthcare providers, due to both physical and mental problems [2–4]. In addition, health care providers had to deal with measures taken in healthcare institutions to prevent the spread of the virus, such as visit restrictions, social distancing and wearing personal protective equipment [5, 6].

Health care providers who provided end-of-life care felt limited to provide care according to their standards [4, 7], and they felt that essential medical and nursing care took priority over psychosocial and spiritual care [4, 8–10]. Research also indicates that visit restrictions resulted in less personalized care, as health care providers were not able to familiarize themselves with the needs and preferences of both patients and their relatives, while relatives were not present to help identify the patient’s needs and wishes [8, 11]. The visit restrictions also limited the way in which health care providers could provide psychosocial and spiritual care, while they felt they should replace the relatives to support the patient [11]. Furthermore, health care providers indicated that communication with family was hindered when doing this via (video)call [12]. In addition, communication with patients (and their relatives) was complicated when wearing personal protective equipment, such as facemasks. Health care providers felt that their interactions with patients were less personal and found it more difficult to make themselves heard or understood [7, 8, 11, 13, 14]. Additionally, in situations of (expected) shortage of personal protective equipment health care providers were not able to visit patients as frequently or for as long as usual and this impacted their care [11].

Literature about end-of-life care during the pandemic mostly consists of cross-sectional designs about the first months of the pandemic. Furthermore, there is a lack of knowledge about to what extent health care providers experienced that end-of-life care was limited over the course of the pandemic, after those first months, and how the COVID-related factors (e.g. visit restrictions) impacted this care and if how this evolved over time in different settings. This study aims to describe to what extent different aspects of end-of-life care (medical, nursing, psychosocial and spiritual care) were limited due to the COVID-19 pandemic throughout the first 18 months of the pandemic according to health care providers. It also aims to assess the association between limitations in care and COVID-19 related circumstances of care (restrictions regarding visits and post-death care and, a scarcity of personal protective equipment) and providers’ characteristics (setting and profession). It is important to reflect on end-of-life care during the course of the pandemic and to what extent it was limited across different domains. This helps us understand what could happen to end-of-life care when we face a new health care crisis. Especially now, as we are heading towards a health crisis due to the shortage of healthcare workers, health care providers may not be able to provide the end-of-life care they are used to, which was also the case during the COVID pandemic.

## Methods

### Design

An observational longitudinal online survey study was conducted as part of the CO-LIVE study. CO-LIVE is a mixed methods study into the experiences of bereaved relatives and health care providers that provided end-of-life care during the COVID-19 pandemic (for both COVID and non-COVID patients) [5]. The aim of the CO-LIVE study is to support clinical practice in finding the right balance between ensuring that care is safe and addressing major public health interests on the one hand, and providing care that is humane and addressing the needs of dying patients and their relatives on the other. In a previous part of the CO-LIVE study among health care providers at the beginning of the pandemic characteristics of patients who died during the pandemic and characteristics of end-of-life care were described [5].

### Population & data collection

Data was collected from a convenience sample of health care providers that provided end-of-life care (for both COVID and non-COVID patients) during the first 18 months of the COVID pandemic (March 2020 – September 2021) with different professions, working in different settings in the Netherlands.

**Fig. 1:**
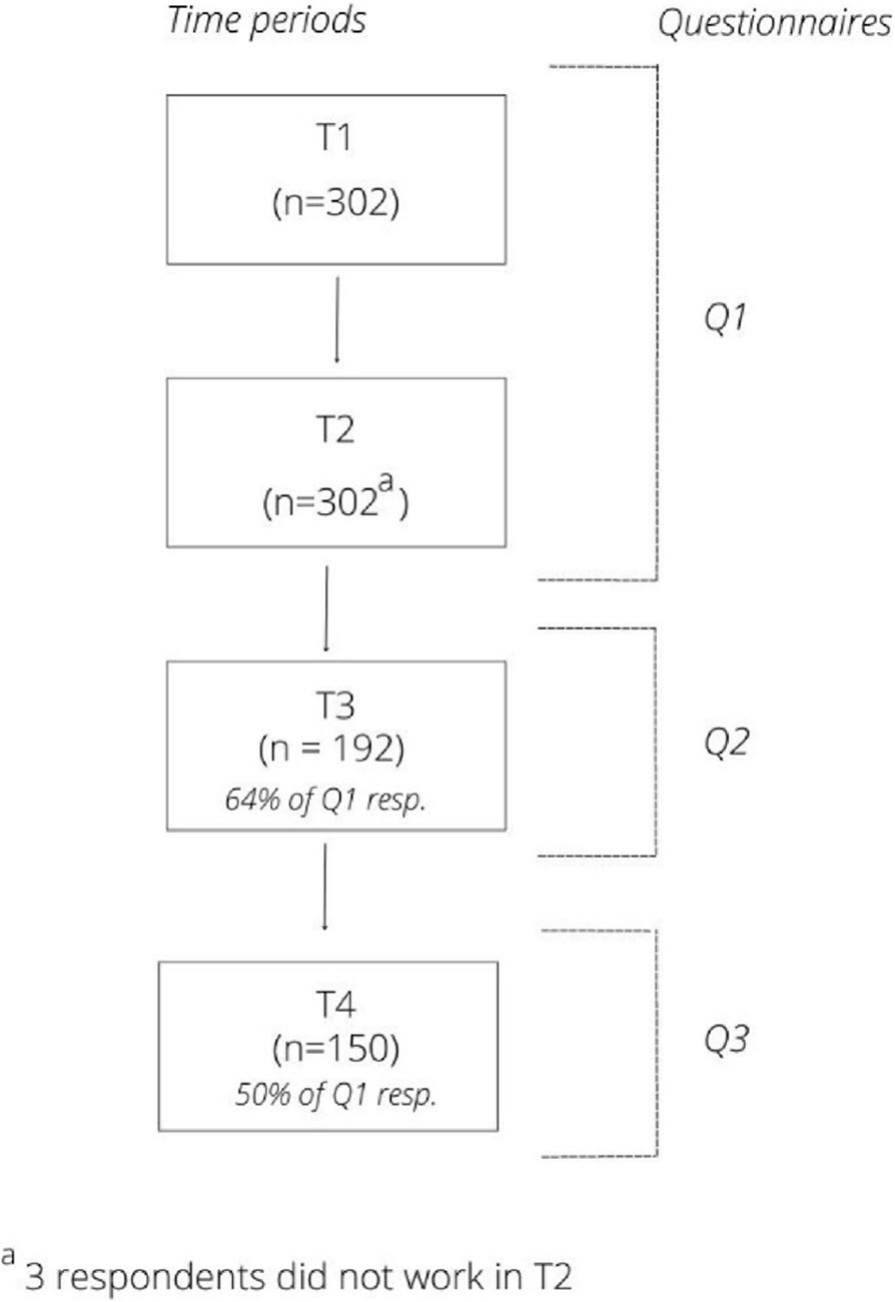
Respondents per time period and questionnaire

**Fig. 2:**
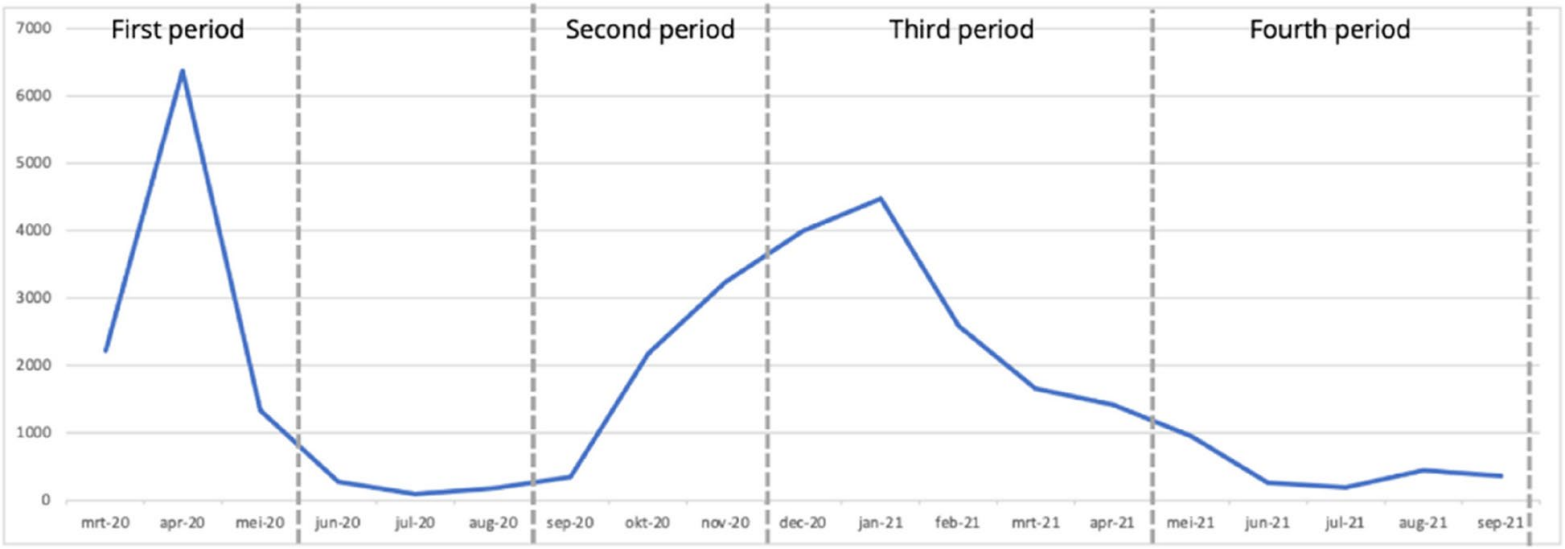
COVID-19 deaths in the Netherlands, march 2020 – September 2021

Data collection covered four time periods, with three questionnaires, Q1, Q2 and Q3 (Fig. 1). Q1 was distributed in November 2020 and questions concerned the periods March 2020 – May 2020 (T1) and September 2020 to November 2020 (T2). These periods are considered to be the first (T1) and (first phase of the) second wave (T2) of the COVID-19 pandemic in the Netherlands [15, 16]. Q2 was distributed in April 2021 and concerned the period between December 2020 – April 2021 (T3). Q3 was distributed in September 2021 and concerned the time period between May 2021 and September 2021 (T4). Figure 2 shows the number of people that died of COVID-19 in the Netherlands within the four time periods [17]. This provides context about the severity of the pandemic in these researched time periods.

Invitations for questionnaire 1 (Q1) were sent to end-of-life care providers that had participated in an previous part of the CO-LIVE study and gave consent to be contacted for further research [5]. Additional respondents for Q1 were recruited via (social) media. When respondents indicated in the Q1 questionnaire that we could approach them for another questionnaire, they received an invitation to Q2 and Q3. No other respondents were recruited for Q2 and Q3. Furthermore, respondents of Q1 who missed Q2 could participate in Q3.

### Measurements

Respondents answered questions about their experiences with end-of-life care provided in each time period during the COVID-19 pandemic. In the questionnaire, end-of-life care was defined as the care provided from the moment it is expected that the patient will pass away in the foreseeable future. This can be days or weeks. Questions about their experiences were self-developed and based on the situation regarding the pandemic and interviews with health care providers about end-of-life care during the pandemic (Appendix 1). All questionnaires were designed and distributed via Survalyzer.

We included questions about different aspects of end-of-life care: medical care, nursing care (such as providing medication and making sure someone is comfortable in bed), psychosocial care and spiritual care (support for important personal beliefs). Health care providers were asked to what extent these different aspects of end-of-life care were limited due to COVID-19 pandemic on a 5-point scale. Answer options were dichotomized to ‘not limited’ (i.e. not limited and slightly limited) and ‘limited’ (i.e. quite limited, very limited and extremely limited). The aspects of care (medical, nursing, psychosocial and spiritual) are based on discussed topics in the validated ‘Care Of the Dying Evaluation’ (CODE™) questionnaire [18].

Characteristics of respondents included gender, age, profession and setting. Setting was categorized into: home, nursing home, hospice facility, other (including for example a GP practice or institutions for people with intellectual disabilities) and multiple settings. Profession was divided in three categories; nurses (including registered nurses, nursing aids and nurse practitioners), physicians (e.g. general practitioners, pulmonary and geriatric physicians) and other (e.g. spiritual counselors, paramedics and volunteers).

The circumstances of care related to COVID-19 consisted of three different variables; visit restrictions, the availability of personal protective equipment and restrictions in post-death care. Visit restrictions (when it became clear that the patient was nearing death) were dichotomized to ‘yes’ for any type of visiting restrictions (maximum persons allowed, maximum time for visits) and ‘no’ for none. Health care providers were asked if there were restrictions in providing post-death care (e.g. not being allowed providing post-death personal care or when patients’ bodies were taken away immediately after death) (yes/no). We asked health care providers if there was enough personal protective equipment available and dichotomized the answers to yes (‘yes’) and no (‘no’ ‘no, not always’, ‘no not enough for everyone who needed it’). This item was not included in Q3, since there was no longer a national shortage of personal protective equipment in T4. We included the item on T4 in the analysis and indicated that there were no shortages.

### Analysis

IBM SPSS statistics 28 and Stata 17 were used to analyze the data. Characteristics from health care providers and COVID-19 related circumstances of care were described to summarize the data per time period. Differences between (the limitation of) the aspects of care within time were analyzed using Chi-square tests.

Generalized Estimating Equations (GEE) were used to study differences between time periods and to investigate what COVID-19 related circumstances of care and characteristics of respondents (as independent variables) are associated with (the limitation of) the different aspects of care due the COVID-19 pandemic (dependent variables). The GEE accounted for clustering of within-subject data (up to four measurements over time per individual). A univariate analysis was done with all independent variables. When independent variables were associated with the dependent variable (*p* < 0.10), the variables were entered in the multivariable regression analysis. All analyses were corrected for age and gender of the health care providers.

### Ethics

Informed consent was obtained from all participants involved in the study. The Medical Research Ethics Committee of the Erasmus MC in Rotterdam, the Netherlands determined exception from formal review under Dutch law (MEC-2020-0254).

## Results

### Characteristics of healthcare providers

Data of 302 (T1), 299 (T2), 192 (T3) and 150 (T4) respondents is included (Fig. 1). The characteristics of the respondents in the different time periods are described in Table 1. Most respondents were women (87.2 – 89.2%) and between 46–60 years of age (44.9—55.8%). Over half of the respondents had a nursing background (61.6 – 71.8%) and most of the respondents worked in a hospital (27.4 –30.4%).

**Table 1:** Characteristics of health care providers during four different time periods (absolute numbers and percentages)

	**T1**	**T2**	**T3**	**T4**
	**Q1** *N* = 302 N (%)		**Q2** *N* = 192 N (%)	**Q3** *N* = 150 N (%)
**Gender**				
Men	32 (10.8)		19 (9.9)	19 (12.8)
Women	265(89.2)		173 (90.1)	129 (87.2)
**Age**				
⩽35 years	61 (20.7)		30 (15.9)	13 (8.8)
36–45 years	58 (19.7)		29 (15.3)	23 (15.6)
46–60 years	132 (44.9)		96 (50.8)	82 (55.8)
> 60 years	43 (14.6)		34 (18.0)	29 (19.7)
**Profession**				
Nurse	216 (71.8)		129 (68.8)	90 (61.6)
Physician	40 (13.2)		24 (12.8)	22 (15.1)
Other	45 (15.0)		35 (18.6)	34 (23.4)
**Setting**				
Home	47 (15.8)		33 (17.6)	20 (13.7)
Nursing home	64 (21.5)		34 (18.1)	29 (19.9)
Hospital	90 (30.3)		53 (28.2)	40 (27.4)
Hospice facility	54 (18.2)		38 (20.2)	29 (19.9)
Other	17 (5.7)		13 (6.9)	15 (10.3)
Multiple	25 (8.4)		17 (9.0)	13 (8.9)

Number of missings range (over Q1-Q3): gender (0–5), age (3–8), profession (0–4), setting (3–5)

### COVID-19 related circumstances of care

Visit restrictions became less present over time (from 91.1% in T1 to 56.3% in T4) (Table 2). The percentage of health care providers that did not have enough personal protective equipment was especially high in the first period (T1: 52.3%), but declined quickly in the next time periods. Over time, more health care providers experienced no restrictions in post-death care (T1: 18.9%; T4: 72.6%). For all COVID-related characteristics the difference between T1 and T4 is significant (Chi-square test: *p* < 0.001).

**Table 2:** COVID-19 related circumstances of care (absolute numbers and percentages)

	**T1** *N* = 302 N(%)	**T2** *N* = 299 N (%)	**T3** *N* = 192 N (%)	**T4** *N* = 150 N(%)
**Visit restrictions**				
Visit restrictions in place	275 (91.1)	246 (82.3)	152 (80.9)	80 (56.3)
No visit restriction in place	27 (8.9)	53 (17.7)	36 (19.1)	62 (43.7)
**Enough personal protective equipment**				
Enough personal protective equipment available	144 (47.7)	279 (93.3)	177 (94.1)	150 (100)
Not enough personal protective equipment available	158 (52.3)	20 (6.7)	11 (5.9)	0 (0.0)
**Restrictions in providing post-death care**				
Allowed to provide post-death care	243 (80.5)	261 (87.3)	170 (90.4)	141 (96.6)
Not allowed to provide post-death care	59 (19.5)	38 (12.7)	18 (9.6)	5 (3.4)

Number of missings range (over T1-T4): visit restrictions (0–8), enough personal protective equipment (0–4), restrictions in providing post-death care (0–4)

### Care provision was limited due to the pandemic

**Fig. 3:**
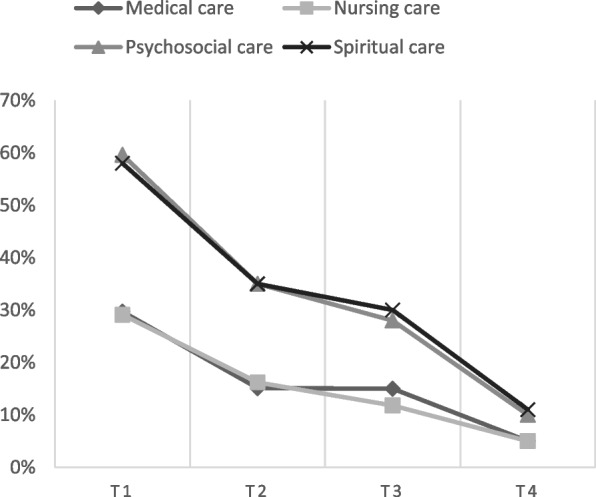
Aspects of end-of-life care that were limited due to the pandemic according to health care providers, over time (percentages)

The percentages of health care providers that considered different aspects of care were limited due to the COVID-19 pandemic in the four time periods is shown in Fig. 3 and Table 3. The percentage of health care providers that indicated that care was limited due to COVID-19, declined over time for all aspects of care: medical care (T1: 29.7% to T4: 5.3%), nursing care (T1: 29.1% to T4: 5.3%), psychosocial care (T1: 59.7% to T4: 10.4%) and spiritual care (T1: 57.7% to T4: 11.4%). In every time period, psychosocial and spiritual care were significantly more often considered limited compared to medical and nursing care (Chi-square test: *p* < 0.001).

**Table 3:** Extent to which aspects of end-of-life care were limited due to the pandemic according to health care providers in four time periods (percentages with 95% confidence intervals)

	**T1**	**T2**	**T3**	**T4**
	**% (95%CI)**	**% (95%CI)**	**% (95%CI)**	**% (95%CI)**
**Medical care**	29.7 (24.6 – 35.1)	14.6 (10.9 – 19.1)	14.9 (10.3 – 20.6)	5.3 (2.4 – 10.1)
**Nursing care**	29.1 (24.1–34.5)	15.8 (11.9 – 20.4)	11.6 (7.4 – 17.0)	5.3 (2.4 – 10.1)
**Psychosocial care**	59.7 (53.9 – 65.2)	34.9 (29.5 – 40.6)	27.8 (21.6 – 34.8)	10.4 (6.1 – 16.3)
**Spiritual care**	57.7 (51.9 – 63.4)	34.9 (29.4 – 40.8)	30.1 (23.6 – 37.2)	11.4 (6.8 – 17.6)

Number of missings range: T1 (12–18), T2 (8–20), T3 (6–14), T4 (6–9)

### Characteristics that are associated with experiencing care as limited

Results of the GEE are shown in Table 4. When compared to the homecare setting, the odds of experiencing medical care as limited were significantly lower in hospice facilities (OR 0.40); the odds of health care providers considering nursing care as limited were higher in hospitals (OR 3.97) and settings in the category ‘other’ (OR 4.27); and the odds of considering psychosocial care and spiritual care as limited were higher in nursing homes (resp. OR 4.54 and 3.10), in hospitals (resp. OR 3.81 and 2.87) and in other settings (resp. OR 4.52 and 2.59). Health care providers that worked in multiple settings were also more likely to experience psychosocial care as limited when compared to homecare (OR 2.96).

**Table 4:** Associations between characteristics of health care providers and care and the four aspects of care being limited due to the pandemic according to health care providers (Odds ratio’s and 95% intervals)^a^)

	**Medical care**		**Nursing care**		**Psychosocial care**		**Spiritual care**	
	Univariate	Multivariate	Univariate	Multivariate	Univariate	Multivariate	Univariate	Multivariate
**Profession**								
Nurse	1.00		1.00		1.00		1.00	1.00
Physician	1.02 (0.55 – 1.92)		1.74 (0.90 – 3.36)		1.12 (0.66 -1.95)		**1.62 (0.98 – 2.68)**	1.42 (0.84 – 2.41)
Other	0.70 (0.39 – 1.27)		0.87 (0.46 -1.65)		0.15 (0.74 – 1.80)		1.22 (0.73 – 2.03)	1.05 (0.62 – 1.76)
**Setting**								
Home	1.00	1.00	1.00	1.00	1.00	1.00	1.00	1.00
Nursing home	1.13 (0.54 – 2.35)	1.05 (0.50 – 2.19)	1.63 (0.57 – 4.62)	1.42 (0.49 – 4.06)	**5.01 (2.29 – 10.94)**	**4.54 (2.08 – 9.87)**	**3.82 (1.84 – 7.91)**	**3.10 (1.59 – 6.34)**
Hospital	1.31 (0.63 – 2.71)	1.45 (0.71 – 2.95)	**3.75 (1.42 – 9.94)**	**3.97 (1.56 – 10.08)**	**3.68 (1.72 – 7.91)**	**3.81 (1.84 – 7.91)**	**3.01 (1.45 – 6.26)**	**2.87 (1.42 – 5.78)**
Hospice facility	**0.40 (0.17 – 0.94)**	**0.42 (0.18 – 0.99)**	0.56 (0.17 – 1.79)	0.63 (0.20 – 1.98)	0.86 (0.37 – 2.00)	0.90 (0.39 – 2.07)	0.74 (0.35 – 1.59)	0.71 (0.34 – 1.46)
Other	1.72 (0.76 – 3.88)	1.67 (0.75 – 3.71)	**4.01 (1.46 – 11.04)**	**4.27 (1.58 – 11.58)**	**4.76 (2.05 – 11.07)**	**4.52 (1.95 – 10.49)**	**3.28 (1.47 – 7.34)**	**2.59 (1.13 – 5.91)**
More than two	0.84 (0.32 – 2.24)	0.81 (0.31 – 2.16)	1.98 (0.56 – 7.04)	1.90 (0.53 – 6.82)	**3.10 (1.31 – 7.36)**	**2.96 (1.25 – 7.01)**	**2.01 (0.88 – 4.59)**	1.67 (0.73 – 3.78)
**Visit restrictions**								
Visit restrictions in place	1.00		1.00	1.00	1.00	1.00	1.00	1.00
No visit restriction in place	1.20 (0.69 – 2.09)		**1.87 (1.04 – 3.35)**	1.47 (0.73 – 2.92)	**2.10 (1.33 – 3.29)**	**2.07 (1.31 – 3.28)**	**2.01 (1.27 – 3.19)**	**1.54 (0.91 – 2.63)**
**Enough personal protective equipment**								
Enough available	1.00	1.00	1.00	1.00	1.00	1.00	1.00	1.00
Not enough available	**1.70 (1.05 – 2.75)**	**1.86 (1.14 – 3.04)**	**1.74 (1.13 – 2.67)**	**2.08 (1.25 – 3.47)**	**1.74 (1.15 – 2.63)**	**2.07 (1.31 – 3.28)**	**1.84 (1.20 – 2.81)**	**2.09 (1.30 – 3.35)**
**Restrictions in providing post-death care**								
Allowed to provide post-death care	1.00	1.00	1.00	1.00	1.00	1.00	1.00	1.00
Not allowed to provide post-death care	**1.74 (1.12 – 2.70)**	**1.71 (1.07 – 2.73)**	**1.59 (1.03 – 2.43)**	1.51 (0.94 – 2.91)	**2.04 (1.43 – 2.92)**	**1.77 (1.19 – 2.63)**	**2.59 (1.79 – 3.76)**	**2.41 (1.61 – 3.61)**
**Time**								
**1**	**6.17 (2.96 – 12.86)**	**3.64 (1.60 – 8.32)**	**6.25 (7.42 – 25.70)**	**3.70 (1.58 – 8.70)**	**13.81 (7.42 – 25.70)**	**8.21 (3.95 – 17.07)**	**10.94 (6.07 – 19.70)**	**5.69 (2.81 – 11.52)**
**2**	**2.68 (1.31 – 5.50)**	**2.19 (1.02 – 4.68)**	**4.67 (2.50 – 8.73)**	2.05 (0.88 – 4.77)	**4.67 (2.50 – 8.73)**	**3.77 (1.91 – 7.50)**	**4.12 (2.30 – 7.38)**	**3.16 (1.64 – 6.08)**
**3**	**2.74 (1.31 – 5.75)**	**2.29 (1.02 – 5.11)**	**3.71 (1.94 – 7.11)**	1.71 (0.65 – 4.46)	**3.71 (1.94 – 7.11)**	**2.96 (1.43 – 6.10)**	**3.52 (1.97 – 6.28)**	**2.65 (1.35 – 5.20)**
**4**	1.00	1.00	1.00	1.00	1.00	1.00	1.00	1.00

Bold values in the univariate analyses indicate OR’s that significantly differ from 1.00 (*p* < 0.1)

Bold values in the multivariate analyses indicates OR’s that significantly differ from 1.00 (*p* < 0.05)

^a^N = up to four observations from 302 respondents; based on Generalized Estimating Equation

Personal protective equipment shortage was significantly associated with considering all aspects of care as limited, (from OR 1.74 for nursing care to OR 2.09 for spiritual care). Furthermore health care providers had a higher odds of experiencing the psychosocial and spiritual care as limited in case of visit restrictions in place (OR 2.07 and 1.54, resp.). When post-death care was restricted, health care providers had a higher odds of experiencing, medical, psychosocial and spiritual care as limited (OR 1.71, 1.77 and 2.41 resp.).

In the multivariable analysis, that takes into account the restrictions per period, health care providers had significantly higher odds of experiencing all aspects of care as limited in T1 (from OR 3.64 for medical care to OR 8.21 for psychosocial care). For medical, psychosocial and spiritual care, there was a significant difference between T2 and T4 (from OR 2.19 for medical care to OR 3.77 for psychosocial care) and T3 and T4 (from OR 2.29 for medical care to OR 2.96 for psychosocial care).

## Discussion

This longitudinal study provides new insights into the extent to which the COVID-19 pandemic limited different aspects of care at the end of life up until 18 months after the start of the pandemic in multiple Dutch healthcare settings, and how COVID-19 related care circumstances impacted care during the pandemic. Especially in the first wave a substantial amount of health care providers considered end-of-life care as limited in all aspects, particularly in the psychosocial and spiritual aspects. This distinction between medical care and nursing care versus psychosocial and spiritual care stayed notable throughout all time periods. Over time, health care providers perceived care as less limited to the point where they barely considered care limited in the last period. Regardless of time period, health care providers experienced end-of-life care as limited when confronted with personal protective equipment shortages, visit restrictions or restrictions in post-death care. Psychosocial and spiritual care were more frequently perceived as limited by health care providers working in nursing homes and hospitals (compared to health care providers working in home care), and by health care providers that were facing restrictions regarding visits and post-death care.

### The COVID-19 related circumstances of care impacted care throughout the pandemic

The longitudinal analyses showed that throughout the first 18 months of the pandemic, family visits restrictions, personal protective equipment shortage and post-death care restrictions impacted end-of-life care.

During shortages of personal protective equipment, all aspects of care were more frequently considered limited. This could be attributed to the conservation of personal protective equipment during shortages, which resulted in health care providers being unable to visit (suspected) COVID-patients as frequently as usual [4, 11]. This could have not only impacted the physical care, but also the social aspects of care. When health care providers were restricted in providing post-death care, they considered the psychosocial and spiritual care more often as limited. This could imply that handling patients' bodies, which often has a ritualistic and cultural meaning for health care providers and families, is also important for the social aspects of care. A study on the quality of palliative care that compared care before and after the pandemic in 2022, found that a culturally sensitive and dignified treatment of the body was less often fully achieved and suggests that this could partly be explained by the (dehumanizing) way bodies were treated during the pandemic [19]. Accordingly, health care providers in our study may have felt that the post-death care was not humane for their standards.

Moreover, visit restrictions impacted psychosocial and spiritual care throughout the pandemic. Previous research showed that health care providers felt that patients relied more on them for emotional support, since their family was limited in their visits [11, 20]. However, visit restrictions also complicated the provision of appropriate emotional support as health care providers had less knowledge of their patient since family could not communicate their wishes to the health care providers due to those restrictions [4, 8, 11].

Thus, the visit restrictions and personal protective equipment scarcity impacted care at the end of life. Especially in the beginning of the pandemic, there was a focus on the prevention of the spread of COVID-19 (and therefore safe physical care), instead of good end-of-life care in all domains [4].

Furthermore, our data shows that over time there were less restrictions regarding post-death care and visits and enough personal protective equipment, which may partly explain why health care providers indicated that care was less limited over time. However, even when taking into account for COVID-19 related circumstances of care in the multivariable analyses, limited care was still experienced in all time periods. This might indicate that that not only the COVID-19 related circumstances of care were subject to change, but also the health care providers themselves. They might have learned how to adapt to the situation, to be flexible with the restrictions and to find solutions for their problems. This is in line with a Scandinavian study about visit restrictions in the Intensive Care Unit [21].

### Psychosocial and spiritual care more often considered as limited (compared to nursing and medical care)

Throughout the first 18 months of the pandemic, health care providers experienced psychosocial and spiritual care more frequently as limited compared to medical and nursing care. The previously mentioned restrictions regarding family visits, personal protective equipment and post-death care could account for some of that difference. However, in addition, in nursing homes and hospitals, both psychosocial and spiritual care were more often considered limited compared to home care. Between these settings and home care, there were differences in what restrictions were at place and how health care providers dealt with these restrictions. In home care, the health care providers, family and patient could collaboratively decide on appropriate preventative measures, while in nursing homes and hospitals, decisions regarding restrictions were made for health care providers, families and patients without their direct input. However, literature suggests more explanations for why psychosocial and spiritual care were (more often) limited. First, health care providers experienced a high work pressure due to different reasons (more patients, less health care providers, time spent on prevention) which lead to prioritizing medical care over psychosocial or spiritual care [3, 4, 8, 20]. Furthermore, the measures (social distancing and wearing personal protective equipment) often limited health care providers in communicating and developing relationships with patients and how it felt distant to provide care is this manner [7, 11]. Additionally, health care providers that did not provide nursing or medical care (e.g. spiritual counselors and social workers), were often banned from the care facilities (especially during the first period) [4, 20]. Diego-Cordero et al., described that nurses were responsible for providing spiritual care due to a lack of specialized care professionals, while often feeling they lacked appropriate training to provide this type of care [20]. According to the Dutch spiritual care guideline, nurses should refer patients to spiritual counselors or specialized nurses in more complex [22], while in the COVID-19 pandemic complex situations were likely to occur more.

### Strengths and limitations

A strength of this study is that it provides a broad overview on end-of-life care during the pandemic, since we looked at multiple healthcare settings and multiple professions, and especially due to the longitudinal design. A limitation of this study is that we did not make a distinction between care for COVID patients and non-COVID patients. There might be differences between these two groups, although most restrictions were applicable to both groups. In addition, we focused on end-of-life care and did not investigate more general factors that may have been experienced as limiting care, such as work pressure. Furthermore, recall bias might have occurred since the questionnaire about the first period was sent during the second period. However, the first wave was such an exceptional time that we expect respondents could remember this period well.

## Conclusion

The COVID-19 pandemic impacted end-of-life care throughout the first 18 months of the pandemic, especially in the first months and on psychosocial and spiritual care, according to health care providers. Restricting family visits and post-death care and personal protective equipment shortage are part of the explanation why health care providers experienced care as limited during the pandemic. However, these do not fully explain why care became less limited over the course of the pandemic. It seems that health care providers found ways to deal with the situation during the pandemic and invented ways to do their work without compromising too much on the quality of care. Health care providers should therefore be involved in decision-making when it comes to (prioritizing) measures in a health crisis. These reflections are important as they show us where priorities lie in times of crisis and what role health care providers could play in maintaining quality care in these situations. This holds true even in new and current health crises, where care may differ from what is considered good quality of care.

## Data Availability

The datasets used and/or analysed during the current study are available from the corresponding author on reasonable request.
